# A Selective Review of the Excitatory-Inhibitory Imbalance in Schizophrenia: Underlying Biology, Genetics, Microcircuits, and Symptoms

**DOI:** 10.3389/fcell.2021.664535

**Published:** 2021-10-21

**Authors:** Yi Liu, Pan Ouyang, Yingjun Zheng, Lin Mi, Jingping Zhao, Yuping Ning, Wenbin Guo

**Affiliations:** ^1^National Clinical Research Center on Mental Disorders and Department of Psychiatry, The Second Xiangya Hospital, Central South University, Changsha, China; ^2^Department of Psychiatry, The Affiliated Brain Hospital, Guangzhou Medical University, Guangzhou, China; ^3^The First School of Clinical Medical University, Guangzhou, China

**Keywords:** schizophrenia, excitatory-inhibitory imbalance (E-I imbalance), glutamate and gamma-aminobutyric acid (GABA) neurotransmissions, genetic models, microcircuits

## Abstract

Schizophrenia is a chronic disorder characterized by specific positive and negative primary symptoms, social behavior disturbances and cognitive deficits (e.g., impairment in working memory and cognitive flexibility). Mounting evidence suggests that altered excitability and inhibition at the molecular, cellular, circuit and network level might be the basis for the pathophysiology of neurodevelopmental and neuropsychiatric disorders such as schizophrenia. In the past decades, human and animal studies have identified that glutamate and gamma-aminobutyric acid (GABA) neurotransmissions are critically involved in several cognitive progresses, including learning and memory. The purpose of this review is, by analyzing emerging findings relating to the balance of excitatory and inhibitory, ranging from animal models of schizophrenia to clinical studies in patients with early onset, first-episode or chronic schizophrenia, to discuss how the excitatory-inhibitory imbalance may relate to the pathophysiology of disease phenotypes such as cognitive deficits and negative symptoms, and highlight directions for appropriate therapeutic strategies.

## Introduction

Schizophrenia is a chronic, debilitating disorder characterized by primary symptoms (positive and negative), social and behavioral disturbances, and cognitive deficits. Interestingly, cognitive deficits often persist even after primary symptoms are relieved by drug treatment. Human and animal studies in the last decades have identified glutamate and gamma-aminobutyric acid (GABA) neurotransmission disturbances as affected factors involved in cognitive deficits of psychiatric disorders. Exploring the brain alterations that contribute to cognitive deficits will shed light on the mechanisms underlying the disease and may help find novel and powerful therapeutic strategies.

## Definition and History of the Excitatory-Inhibitory Balance Hypothesis

The majority of neurons in the neocortex are glutamatergic excitatory pyramidal neurons (Somogyi et al., 1998; Sugino et al., 2006), while around 20% of the cortical neurons are GABAergic inhibitory interneurons (Tatti et al., 2017). Neural information consists of both excitatory signals from glutamatergic pyramidal neurons and inhibitory signals originate from GABAergic interneurons. In a healthy brain, excitatory and inhibitory information is perfectly balanced through the control by multiple developmental processes. Maintenance of the balance of excitatory and inhibitory signals is critical for the development and function of cortical microcircuits and neural networks. Disruption of this finely tuned excitatory-inhibitory (E-I) balance has been proposed as a hypothesis providing insights into the pathomechanisms underlying neurodevelopmental and neuropsychiatric disorders (Rubenstein and Merzenich, 2003; Yizhar et al., 2011; Lisman, 2012).

In general, the relatively simple conceptualization of E-I balance always refers to the balanced state of the overall excitatory and inhibitory levels of singular entities at both single cell and global circuit levels (Rubenstein and Merzenich, 2003). Recently, Sohal and Rubenstein (2019) have clarified the multidimensional concept of this balance, stating that excitation and inhibition signals originate from multiple sources and can act on different targets. Furthermore, the excitatory-inhibitory balance may contribute to the balanced activity of different subtypes of excitatory or inhibitory neurons, rather than the simple ratio between the overall level of excitation and inhibition (Sohal and Rubenstein, 2019). The multidimensional concept of E-I balance highlights the essentiality of understanding of the multifaceted mechanisms that can result in E-I imbalance and contribute to the pathophysiology of the disease. Imbalanced excitatory and inhibitory information could contribute to disturbance of higher cognitive functions, such as sensory and working memory processing (Rao et al., 1999; Casanova et al., 2007; Opris and Casanova, 2014). Understanding the disturbance of the excitatory-inhibitory balance can provide more insight into the mechanisms underlying neurodevelopmental disorders (NDDs) such as schizophrenia, autism spectrum disorders, intellectual disability, and epilepsy, and further provides guidance for future therapies for these NNDs.

## Dysfunction of GABAergic and Glutamatergic Neurotransmission in Schizophrenia

The alteration of either GABA or glutamate levels contribute to an abnormal GABA/glutamate ratio, which further strengthens the proposition of an E-I imbalance in schizophrenia.

### Evidence for GABAergic Dysfunction in Schizophrenia

#### Post-mortem Cerebrospinal Fluid and *in vivo* Plasma Levels

Postmortem studies in human subjects have demonstrated altered cerebrospinal fluid (CSF) levels of gamma-aminobutyric acid in several psychiatric diseases, including schizophrenia (Lichtshtein et al., 1978; Van Kammen et al., 1982), depression (Gold et al., 1980; Gerner and Hare, 1981), bipolar disorder, and anorexia nervosa (Gerner and Hare, 1981). Lower GABA levels in the CSF of schizophrenic patients than healthy subjects were first reported over 40 years ago (Lichtshtein et al., 1978), and were further found to be significantly reduced in female schizophrenic patients during the early years of the illness (Van Kammen et al., 1982). Lower GABA levels may also be present in the plasma, albeit the non-significant difference found in a previous study (Petty and Sherman, 1984). GABA levels have been shown to increase with age, duration of illness, and further increase with long-term neuroleptic treatment (Van Kammen et al., 1982).

#### Imaging Gamma-Aminobutyric Acid Levels *in vivo*

Imaging studies quantifying GABA levels via proton magnetic resonance spectroscopy (^1^H-MRS) have provided evidence of alterations in GABA concentration in several brain regions. While *in vivo* GABA levels were first assessed in the first-episode, antipsychotic-naive male patients with schizophrenia by Stanley et al. over 20 years ago, the original findings showed no significant changes in the left dorsolateral prefrontal cortex (DLPFC), as compared to healthy controls (Stanley et al., 1994). On the contrary, a 25% elevation in the ratio of GABA and glutamate to creatine was reported in the right prefrontal cortex of medication-free patients with first-episode schizophrenia (Choe et al., 1994), indicating an increased prefrontal glutamatergic and GABAergic activity. Consistently, a 15.5% elevation of the GABA/creatinine ratio in the anterior cingulate cortex (ACC) and a 11.9% elevation of the GABA/creatinine ratio in the parieto-occipital cortex (POC) have been found, respectively, in patients with chronically treated for schizophrenia (Ongür et al., 2010). Kegeles et al. (2012) reported a 30% increase in GABA levels in the medial prefrontal cortex (mPFC) but not the DLPFC in unmedicated patients with first-episode schizophrenia, suggesting distinct alterations in the excitatory-inhibitory balance in specific brain regions. Although systematic reviews and meta-reviews across psychiatric disorders indicated no statistical difference in levels of GABA in schizophrenic patients compared to controls (Schür et al., 2016), recent studies using advanced ^1^H-MRS technology have provided a new insight into *in vivo* GABA concentrations in patients with schizophrenia by reporting a tendency to reduce *in vivo* ACC GABA levels in chronic schizophrenia patients in older age (Rowland et al., 2013), and a significant reduction of *in vivo* GABA levels in patients with first-episode schizophrenia (Chiu et al., 2018). In addition, these reports indicated that decreased *in vivo* GABA levels was correlated with the severity of illness (indicated by total and general PANSS performance), and might be associated with poor attention in medication-naive patients with first-episode schizophrenia (Orhan et al., 2018). More recently, 7T ^1^H-MRS studies also provided evidence that patients with first-episode schizophrenia exhibited reduced ACC (Wang et al., 2019) and cortical GABA levels (Thakkar et al., 2017), and this reduction is significant when compared either with healthy siblings or the combination of their healthy siblings and participants in the healthy control group (Thakkar et al., 2017).

### Evidence for Glutamatergic Dysfunction in Schizophrenia

#### Glutamate and Glutamate Markers in the Cerebrospinal Fluid, Post-mortem Tissue and Plasma

Human studies have suggested a predominant role in the contribution of corticolimbic glutamatergic neurotransmission dysfunction in schizophrenia. Heterogeneity in the CSF glutamate concentrations in patients with schizophrenia has also been reported. Kim et al. (1980) first reported reduced CSF glutamate levels in schizophrenic patients and proposed the idea that a decreased glutamatergic function might play a role in the etiology of this disorder. Tsai et al. (1995) found diminished glutamate concentrations in the hippocampus of schizophrenic patients. In addition, increased levels of *N*-acetyl-aspartyl glutamate (NAAG), an acidic dipeptide that acts as an NMDA receptor antagonist (Coyle, 1997), was found in the centrum semiovale in schizophrenia patients at younger age (Rowland et al., 2013). However, these findings suggesting lower glutamatergic activity in schizophrenic patients were only replicated by a few subsequent studies (Bjerkenstedt et al., 1985; Macciardi et al., 1990). Conversely, other studies reported increased levels of the cerebral glutamate and glutamate receptor antagonist kynurenic acid (KYNA) in schizophrenia patients treated with antipsychotics (Gattaz et al., 1982; Nilsson et al., 2005). Finally, a meta-analysis including a total of 320 schizophrenic patients and 294 healthy controls showed that peripheral glutamate levels in schizophrenic patients were significantly higher than those in the control (Song et al., 2014).

#### Glutamatergic Neurotransmission and Glutamate Receptors

The glutamatergic system is the major excitatory system linking the cortex, limbic system, and the thalamus. Several studies have proposed a role of glutamatergic neurotransmission dysfunction in the pathophysiology of schizophrenia (Coyle, 1996). Csernansky and Bardgett suggested that schizophrenic symptoms, especially negative symptoms, and cognitive deficits, might be associated with dopaminergic neurotransmission initiated by lesions of limbic-cortical neurons and decreases in glutamatergic inputs to the nucleus accumbens and the ventral striatum (Csernansky and Bardgett, 1998; Goff and Coyle, 2001). Thus, glutamatergic neurotransmission is considered as a promising target for potential antipsychotic drugs (Chiu et al., 2018; Mccutcheon et al., 2020).

The postsynaptic activities of glutamate are mediated by different families of glutamate receptors, including ionotropic receptors such as the *N*-methyl-D-aspartic acid (NMDA) receptor, the α-amino-3-hydroxy-5-methyl-isoxazole-4-proprionic acid (AMPA) receptor and the kainate receptor, and metabotropic receptors coupling to G-proteins which regulate intracellular metabolic processes (Goff and Coyle, 2001). The NMDA receptor (NMDAR) plays a critical role in many molecular and cellular processes modulate synaptic plasticity, neuronal development, and differentiation. In the brains of schizophrenia patients, a disruption of NMDAR expression and localization has been demonstrated (Kristiansen et al., 2007), as well as NMDAR dysfunction in schizophrenia-like behaviors in both humans and animal models (Wang et al., 2004; Guo et al., 2009). Impaired NMDAR function might cause deficits in the PV-positive neuron-mediated inhibition of cortical circuits in schizophrenia (Gonzalez-Burgos and Lewis, 2012; Jadi et al., 2016) and could further mediate cognitive impairments associated with schizophrenia (Karlsgodt et al., 2011). The administration of NMDAR antagonists, such as ketamine, induces schizophrenia-like symptoms, including positive and negative symptoms and cognitive deficits (Thiebes et al., 2017). Similarly, studies have consistently demonstrated a decreased AMPA receptor function in the hippocampus of patients with schizophrenia (Kerwin et al., 1988). Likewise, lower levels of AMPA receptors have been found in the medial temporal lobe of patients with schizophrenia (Meador-Woodruff and Healy, 2000).

## Electrophysiology

Electrophysiology is a most important method to study the E-I balance. The E/I ratio is often determined by the changes in both miniature excitatory postsynaptic currents (mEPSCs) and miniature inhibitory postsynaptic currents (mIPSCs) from pyramidal neurons. Notably, some studies suggested that electrophysiological biomarkers, such as the P3b event-related potentials (ERP), P50 suppression, the gamma band oscillations, mismatch negativity (MMN), and resting state electroencephalography (EEG) characteristics, might be intermediate phenotypes that are predictive of treatment efficacy and pathophysiology elucidation of schizophrenia (Nagai et al., 2013; Light and Swerdlow, 2015; Thiebes et al., 2017). The gamma band oscillations and MMN are two electrophysiological features closely linked to activities of glutamatergic and GABAergic transmission systems.

Mismatch negativity often refers to a negative deflection to the perceived environmental stimuli. Previously, it has been demonstrated that MMN is attenuated in patients with schizophrenia (Erickson et al., 2016; Owens et al., 2016). Progressive MMN amplitude attenuation may be explained by the long-term dysfunction of the glutamatergic NMDAR neurotransmission system, which might be attributed to extensive gray-matter loss in the whole cortex (Coyle, 2006; Salisbury et al., 2007; Rasser et al., 2011; Näätänen et al., 2016). The use of NMDA receptor antagonists, such as ketamine or MK-801, could reduce the MMN amplitude in healthy human subjects (Umbricht et al., 2000). The NMDA receptor subunit genes GRIN1, GRIN2B, and GRIN3B are reported to be associated with the duration MMN as well (Lin et al., 2014).

Gamma band oscillations abnormalities have been consistently reported in schizophrenia studies (Uhlhaas and Singer, 2010). According to these studies, impaired gamma frequency oscillations may contribute to altered inhibition from parvalbumin-containing GABA neurons in schizophrenia (Volk et al., 2016), and reduced gamma band oscillations could result in impairments in working memory, attention and sensory processing in patients with schizophrenia (Gonzalez-Burgos et al., 2015). Thus, deficient gamma oscillations may indicate pathogenic processes in the GABAergic neurotransmission system, which may result in deficiency in inhibitory neurotransmission. Additionally, gamma band oscillations computational models exhibited stronger gamma band oscillations with weakened excitatory to enhanced inhibitory connections, as a consequence of GABAergic hyperfunction and NMDAR system hypofunction in the E-I network (Volman et al., 2011; Jadi et al., 2016).

## Alterations in Genetic and Epigenetic Factors of Glutamate and GABA Neurotransmission Pathways

### Genetic Mechanisms Underlying Altered E-I Balance

In this section, we aim to discuss the genetic causes underlying glutamate and GABAergic synaptic transmission dysfunction in schizophrenia with a focus on the effects of *CYFIP1* and *SYNGAP1*.

Several genes are involved in the glutamate and GABA neurotransmission pathways are altered in schizophrenic patients. The expression of genes implicated in glutamate and GABA neurotransmission pathways was found to be altered across the visuospatial working memory network in layer 3 in subjects with schizophrenia (Hoftman et al., 2018). Generally, gene markers associated with glutamatergic transmission have been found to be disturbed in the visuospatial circuit in schizophrenia. For instance, it was demonstrated that vesicular glutamate transporter-1 (vGLUT1) expression was significantly lower in schizophrenic patients, compared to healthy subjects, throughout the entire visuospatial working memory network, while levels of *GRIN1* transcript were higher in the primary visual cortex in patients with schizophrenia. In addition, the excitatory amino acid transporter (EAAT) 1 and EAAT2 play a key role in modulating glutamatergic activity and have been found to be associated with schizophrenia (Parkin et al., 2018). According to Hoftman et al. (2018), levels of EAAT2 mRNA transcripts was higher in the primary visual cortex and visual association cortex in patients with schizophrenia, as compared to healthy controls. And a reduced level of gene expression involved in GABAergic activity was shown in schizophrenic patients compared to controls. *GAD1*, a gene encoding the GABA synthetic enzyme glutamate decarboxylase 67-kDa (also referred to as gamma aminobutyric acid 67, GAD67), is downregulated in patients with schizophrenia (Fujihara et al., 2015, 2020; Tao et al., 2018), which may contribute to the disinhibition associated with schizophrenia (Volk and Lewis, 2002). This inference was also demonstrated by a study on animals with reduced GAD67 expression in PV-positive interneurons (Zhang et al., 2008). As expected, the expression of GABA transporter GAT1 is reduced in a subset of GABA neurons in the brain of patients with schizophrenia (Volk et al., 2001). Additionally, vesicular GABA transporter (vGAT), a GABAergic associated transcript, has been found to be significantly lower in the posterior parietal cortex in brains with schizophrenia, as compared to healthy brains. Similarly, it was also found that knockdown of syndapin I (synaptic dynamin-associated protein I) led to a reduced intracellular accumulation of overexpressed GluA2, further causing impaired function of glutamatergic AMPAR and NMDAR, and resulting in schizophrenia-like phenotypes (Koch et al., 2020). Lastly, a reduction of GABA in the cortex and hippocampus has been shown to result in schizophrenia-associated negative symptoms (Kolata et al., 2018) and cognitive impairments (Fujihara et al., 2020). Of note, schizophrenia-associated copy number variations (CNVs) are common for genes involved in inhibitory GABAergic and excitatory glutamatergic neurotransmissions (Pocklington et al., 2015).

*CYFIP1* is a key pathogenic gene located on 15q11.2 (De Rubeis et al., 2013; Oguro-Ando et al., 2015). Numerous studies have identified a loss of CNVs on 15q11.2 in individuals with schizophrenia (Rees et al., 2014; Marshall et al., 2017), and polymorphisms and rare variants in *CYFIP1* are also found to be associated with susceptibility to schizophrenia (Yoon et al., 2014). CYFIP1 is enriched in the synapses of excitatory neurons, where it regulates the plasticity and development of dendritic spines (De Rubeis et al., 2013; Pathania et al., 2014). Davenport et al. (2019) have shown that CYFIP is also enriched at the postsynaptic sites of inhibitory neurons. According to Davenport et al. (2019), *CYFIP1* deficiency leads to enhanced inhibitory transmission in glutamatergic neurons *in vivo*, eliciting increased levels of synaptic proteins such as the postsynaptic GABA(A) receptor β2/3-subunits and neuroligin 3, and oversized inhibitory synapses. Conversely, increased expression of CYFIP causes an increase in excitatory synapses on both the shaft and dendritic spines, further disrupting inhibitory synaptic transmission. Collectively, *CYFIP1* dysfunction, both overexpression and deletion, can result in an abnormal function of glutamatergic and GABAergic synaptic transmission, leading to an excitatory-inhibitory imbalance (Davenport et al., 2019).

SYNGAP1 (Synaptic Ras GTPase activating protein 1) is an essential component of the NMDAR complex abundantly expressed in the postsynaptic density (PSD) of excitatory glutamatergic neurons (Chen et al., 1998; Kim et al., 1998) and GABAergic interneurons (Berryer et al., 2016). In humans, *de novo* loss-of-function mutations in *SYNGAP1* are among the most common causes of non-syndromic sporadic intellectual disability (Hamdan et al., 2009) and may be associated with schizophrenia (Xu et al., 2012). *SYNGAP1* is also proposed as one of the top 10 prioritized genes with susceptibility to schizophrenia (Niu et al., 2019). Decreased levels of SYNGAP1 and its interaction partner PSD95 were well-documented in the brains of schizophrenia patients (Funk et al., 2009). And targeted deletion of *SYNGAP1* was found to result in an enhanced membrane excitability and a disturbed excitatory-inhibitory balance (Berryer et al., 2016), which was one of the most common mechanisms underpinning NDDs. Studies have demonstrated that *Syngap1* haploinsufficiency in mice caused profound core features of schizophrenia, such as hyperactivity, decreased prepulse inhibition, impaired working and spatial reference memory (Guo et al., 2009; Nakajima et al., 2019), generalized cortical excitability (Ozkan et al., 2014), and abnormal gamma oscillation (Berryer et al., 2016), whereas supplementation of the Syngap1 protein can reverse or attenuate the manifestation of behavioral problems, cognitive deficits and medically refractory seizures (Vazquez et al., 2004; Aceti et al., 2015).

### Epigenetic Mechanism and RNA Interference Application in Schizophrenia Research

It has also been suggested that epigenetic alterations of genes involved in the GABA and glutamate pathways occur in patients with schizophrenia. According to Guidotti, psychotic patient exhibited increased methylation of promoters of GAD67 in their brains (Guidotti et al., 2011). And lymphocytes of chronic schizophrenia patients exhibited higher *DNMT1* mRNA expression levels and lower *GAD67* mRNA levels than non-psychotic controls (Zhubi et al., 2009). A study also demonstrated that methylation levels of two CpG loci within the putative GAD1 promoter was significantly associated with the expression of GAD25 in the DLPFC and with the SNP rs3749034, a SNP associated with schizophrenia (Tao et al., 2018).

RNA interference (RNAi) is a gene regulation process that mediates sequence-specific gene silencing through functional small inhibitory RNAs (∼21 nt) (Provost et al., 2002). To date, RNAi technology is well established, and served as a powerful molecular tool to study gene functions in biological processes, manipulate *in vitro* and *in vivo* gene expression, and guide new treatment strategies for diseases that lacks effective treatment (Boudreau and Davidson, 2010). The preclinical application of RNAi strategies could target knocked-down genes in glutaminergic and GABAergic neurons and induce disturbance in E-I network in experimental animals. For instance, the role for DISC1 in the development of schizophrenia has been repeatedly reported in previous preclinical animal model studies. Notably, this DICS1 model was generated by using RNA interference (RNAi) approach (Kubo et al., 2010). It was found that *DISC1* deficiency in neurons led to increased GABAergic spontaneous synaptic current frequency and excessive inhibitory inputs from PV-positive GABAergic interneurons, resulting in dysfunction of excitatory glutamatergic synapses as well as disruption of the E-I balance (Delevich et al., 2020). RNAi technologies could also improve drug efficacy. Intranasal application of antipsychotic or antidepressant conjugated siRNAs that targeted corresponding receptors may elicit improvement faster than conventional drug treatment (Artigas et al., 2018).

### Models of Schizophrenia With Altered Excitatory-Inhibitory Balance

Experimental studies have explored the contribution of altered E-I balance in animal models of different neuropsychiatric diseases. Transgenic knock-out (KO) of *FMR1* in the somatosensory cortex of mice is a commonly used model for Fragile-X Syndrome (O’donnell et al., 2017), while a conditional MeCP2 allele KO in GABAergic neurons in mice is considered an adequate model for Rett Syndrome (Chao et al., 2010). Having described the genetic and epigenetic mechanisms that can impact the E-I balance, we now summarize studies that have examined mouse models of schizophrenia to enhance our understanding of alterations to the E-I balance.

#### MK-801 Schizophrenia Model

MK-801, a non-competitive NMDA receptor antagonist, can block NMDAR and introduce hyper-locomotor activity. MK-801 schizophrenia model is a pharmacological model. The hyperactivity induced by MK-801 could model part of the positive symptoms in schizophrenia. Notably, the MK-801 model can represent the most widely applied animal model for positive symptoms in schizophrenia. MK-801 treatment for rats could cause disruption in their recognition memory, reduction in the number of PV-positive interneurons and the upregulation in vGLUT1/vGAT ratio through their whole life (Li et al., 2015; Ma et al., 2020). VGLUT1 and vGAT are cortical E-I biomarkers, and the elevation in vGLUT1/vGAT ratio reflects the upregulation of E/I ratio, which indicates an imbalanced E-I network. Furthermore, it is previously reported that NMDAR antagonists take antipsychotic effect specifically by blocking NMDARs in PV-positive interneurons (Bygrave et al., 2016), and NMDAR hypofunction or deletion in PV-positive interneurons could prevent the hyperactivity induced by NMDAR antagonists (Belforte et al., 2010; Carlén et al., 2012). The hypofunction of NMDAR is closely connected to the function of GABAergic neurons. Repeated treatment with NMDAR antagonists could lead to NMDAR hypofunction and further decreased GAD67 expression in cortical GABAergic neurons, leading to reduced GABAergic activities (Rujescu et al., 2006; Behrens et al., 2007). This result could partially contribute to higher sensitivity to NMDAR antagonists for GABAergic interneurons than pyramidal neurons. Thus, evidence suggested that the prefrontal GABAergic transmission deficiency induced by MK-801 could be relieved by pharmacological treatment with GABAAα1-receptor-positive allosteric modulator (Thomases et al., 2013). In summary, the imbalance of the cortical excitation and inhibition networks indicates a core pathophysiology for schizophrenia.

#### *DISC1* Mouse Model

Disrupted schizophrenia 1 (*DISC1*) is a strong candidate gene for major psychiatric disorders such as schizophrenia and autism spectrum disorders. It interacts with many types of proteins involved in neural development and is linked to numerous cognitive impairments in schizophrenia patients (Roberts, 2007; Teng et al., 2018). *DISC1* knockdown in mature dentate granule neurons drastically reduces the number of mature mushroom spines and decreases the frequency of glutamatergic spontaneous synaptic currents, which indicates deficits of the glutamatergic synaptic transmission. Conversely, *DISC1* deficiency in neurons leads to increased GABAergic spontaneous synaptic current frequency and excessive inhibitory inputs from PV-positive GABAergic interneurons, resulting in dysfunction of excitatory glutamatergic synapses in *DISC1*-deficient neurons (Delevich et al., 2020). The *DISC1* deficiency-induced alterations in GABAergic and glutamatergic synaptic transmission supporting the hypothesis of the E-I imbalance, which then results in cognitive deficits (Kang et al., 2019).

#### *RELN* Mouse Model

The reelin gene (*RELN*) has been proposed as a risk gene implicated in several psychiatric disorders, including schizophrenia (Beasley et al., 2020), depression (Caruncho et al., 2016), and autism spectrum disorders (Aldinger et al., 2011). Reelin modulates neuronal morphology, development, as well as diverse aspects of synaptic plasticity and functions. In various studies, reelin supplementation in adult brains could increase hippocampal synaptic activity (Hethorn et al., 2015), long-term potentiation (Pujadas et al., 2010), and promotes dendrite and spine development (Pujadas et al., 2010; Bosch et al., 2016), consequently enhancing cognitive ability. The Heterozygous reeler mouse (HRM) is a model of schizophrenia. Recent studies have shown altered excitatory synaptic transmission in prefrontal pyramidal neurons and postnatal maturation of the PFC in HRM mice, which is in line with findings in schizophrenia patients (Iafrati et al., 2014). Reduced reelin levels have been further shown to result in an impaired maturation of GABAergic synaptic transmission. Reelin deficiency-induced dysfunction of GABAergic inhibition consequently impacts the synaptic excitatory-inhibitory balance. The reelin deficiency model, therefore, provides a mechanism for the altered E-I balance of prefrontal circuits in the pathophysiology of psychiatric disorders, particularly schizophrenia (Bouamrane et al., 2016).

#### *CLU3* Mouse Model

Cullin 3 (CUL3) is a component of the CUL3-RING E3 ubiquitin ligase complex (Pintard et al., 2004), which regulates a series of cellular functions such as anti-oxidation, the cell cycle, protein trafficking, and signal transduction (Andérica-Romero et al., 2013). *CUL3* mutations are considered risk factors for schizophrenia. Mice with *Cul3* deficiency exhibit anxiety-like behaviors, social deficits, and alterations in both glutamatergic and GABAergic neurotransmission. Dong et al. (2020) found an elevated frequency of both mEPSCs and mIPSCs in CA1 hippocampal pyramidal neurons of *GFAP-Cul3^*f/+*^* mice. Moreover, these mice also presented with an elevated eEPSC/eIPSC and E-I ratios. These changes may underlie the social deficits and abnormal behaviors of *CLU3* mutant mice, suggesting that an E-I imbalance may be the mechanism for eliciting psychotic behaviors and symptoms (Dong et al., 2020).

#### *ATX* Mouse Model

The LPA-synthesizing enzyme autotaxin (ATX) is expressed in the astrocytic compartment of excitatory synapses and modulates glutamatergic transmission, and this regulates cortical E-I balance and controls sensory information processing in mice and humans. ATX inhibition is used for treatment interventions in animal models of psychiatric disorders. As it normalizes cortical hyperexcitability and altered behaviors in a ketamine-induced animal model of schizophrenia, it is considered to be effective treatment strategy for cortical hyperexcitation presented in patients with psychiatric disorders (Thalman et al., 2018).

#### PGC-1α Mouse Model

Peroxisome proliferator activated receptor γ coactivator 1α (PGC-1α) is a transcriptional coactivator found in the hippocampus. It is rich in inhibitory interneurons and regulates parvalbumin transcription. Transcriptional dysregulation of PGC-1α in parvalbumin interneurons causes altered inhibition, which represents a consistent pathophysiological feature of schizophrenia (Lewis and Hashimoto, 2007). Decreased mRNA expression of parvalbumin in interneurons is frequently reported (Reynolds et al., 2004), and genetic deletion of PGC-1α in mice was found to result in decreased protein expression of parvalbumin in interneurons (Lucas et al., 2010). Loss of PGC-1α enhances basal inhibition, including inhibition of parvalbumin interneurons, and contributes to the decrease in the E-I ratio in CA1 pyramidal cells in response to Schaffer collateral stimulation in slices from young adult mice. This reduces the spread of activation in CA1 and seriously limits spiking in pyramidal cells and reduces hippocampal output and gamma oscillations. Eventually, the altered E-I ratio and CA1 output may lead to persistent circuit dysfunction. Taken together, PGC-1α deficiency is highly likely to be associated with psychiatric diseases and may attribute to circuit-dependent alterations in the E-I balance (Bartley et al., 2015).

#### The 22q11.2 1.5 Mb Deletion [*Df(16)A^±^*] Mouse Model

*De novo* heterozygous and recurrent microdeletions of the chromosome 22q11.2 locus account for approximately 1–2% of sporadic cases of schizophrenia, and are considered to be one of the biggest genetic risk factors for this disease (Cantonas et al., 2019). The majority of recurrent 22q11.2 deletion carriers exhibit a 3 Mb chromosomal deficiency, while 7% exhibit a nested 1.5 Mb chromosomal deficiency (Choi et al., 2018). A study showed that the deletion of this region resulted in the loss of 35–60 known genes that might be critical for neurodevelopment (Cantonas et al., 2019). For instance, microdeletion of 22q11.2 could result in *DGCR8* deficiency, leading to altered short-term plasticity in the PFC (Fénelon et al., 2011). The mouse model was established by Stark et al. (2008), which has a 1.5 Mb deletion on chromosome 22q11.2, is also referred to as the *Df(16)A^±^* mouse. *Df(16)A^±^* mice feature deficits in prepulse inhibition and working memory, which are in line with symptoms in patients with schizophrenia (Stark et al., 2008; Fénelon et al., 2013; Xu et al., 2013; Ellegood et al., 2014). It is worth noting that, although *Df(16)A^±^* mice have a normal E-I balance at baseline, they are unable to keep this balance of dopaminergic modulation, resulting in an altered E-I balance with higher excitatory and lower inhibitory transmission during KCNQ2 (Potassium Voltage-Gated Channel Subfamily Q Member 2)-dependent abnormal dopaminergic regulation (Choi et al., 2018).

#### NL2 R215H Knock-in Mouse Model of Schizophrenia

Neuroligin-2 (NL2), a postsynaptic cell adhesion protein predominantly expressed in inhibitory synapses, is required for synapse maturation, specification, and stabilization. The R215H single-point mutation of NL2 has been reported to be associated with schizophrenia (Chen et al., 2017), which was supported by a study showing that a knock-in of NL2 R215H in mice is associated with deficits in pre-pulse inhibition, learning and memory functions. The NL2 R215H knocked-in mice were also found to have a significantly reduced GABAergic inhibitory synaptic transmission (mIPSCs, eIPSCs) and decreased levels of inhibition-related proteins [PV, GABA(A) receptor, vGAT] in the mPFC, which could result in a disrupted E/I ratio and contributed to aberrant gamma oscillation (Chen et al., 2020).

## Excitation-Inhibition-Related Microcircuit Deficits in Schizophrenia

In the following sections, we aim to address specific brain circuits that might account for schizophrenia symptoms.

### The Prefrontal Cortex

Many mental disorders, including schizophrenia, are accompanied by complex cognitive impairments, especially abnormal executive and working memory functions caused by dysfunction of the prefrontal cortex (PFC) (Lisman, 2012). Development of the mammalian PFC is characterized by a prolonged postnatal maturation period during which the PFC remains vulnerable to psychiatric influence. A disrupted E-I balance in PFC pyramidal neurons has been implicated in multiple PFC-dependent behaviors (or their alteration), including cognition, social interaction, and anxiety in psychiatric disorders (Ferguson and Gao, 2018b). In particular, working memory depends critically on coordinated circuitry activity of excitatory pyramidal neurons and subpopulations of inhibitory GABAergic neurons (such as PV-expressing neurons) in the dorsolateral prefrontal cortex (DLPFC), especially in layer 3 of the DLPFC (Hoftman et al., 2017). However, the expression of molecular substrates involved in glutamate and GABA neurotransmission in the layer 3 or the layer 3 local circuit of DLPFC is disrupted in schizophrenia patients, compared with unaffected normal subjects (Dienel et al., 2020). Of note, in an extensively validated spiking network model of spatial working memory, synaptic compensation that help restoring the E-I balance has been shown to ameliorate working memory deteriorations (Murray et al., 2014). Moreover, elevation of the cellular E-I balance within the mPFC has been found to elicit a profound deterioration in cellular information processing, and is associated with specific behavioral dysfunctions in human subjects (Yizhar et al., 2011). In addition, disinhibition of excitatory pyramidal neurons in layer 2/3 of the mPFC has been shown to be accompanied by decreased GABA release from local PV-positive interneurons in *Disc1* Locus Impairment mice, and by disrupted feedforward inhibition in the circuit ranging from the mediodorsal thalamus to the mPFC (Delevich et al., 2020). Similarly, impaired maturation and refinement of glutamatergic and GABAergic synaptic transmissions in the postnatal PFC were found in mice with reelin haploinsufficiency, which ultimately impacted the sequence of synaptic E-I balance development, and therefore, provides a potential mechanism for altered E-I balance in the prefrontal circuit in patients with psychiatric disorders (Bouamrane et al., 2016).

### Hippocampus

It has been recognized that the hippocampus is implicated in schizophrenia due to its altered functional activation in schizophrenia as well as the memory deficits associated with the disease. In healthy participants, higher glutamate and glutamine levels could predict higher levels of functional connectivity from the hippocampus to the anterior default mode network. However, an inverse relationship was observed in medication-free individuals with first-episode psychosis (Nelson et al., 2020). Furthermore, knockdown of S-SCAM, a unique synaptic scaffolding protein that localizes to both excitatory and GABAergic synapses in hippocampal neurons *in vitro*, causes a drastic loss of components in both GABAergic pre- and post-synapses, resulting in an attenuation of GABAergic synaptic transmission. Intriguingly, S-SCAM overexpression weakens GABAergic synapses by leading to a loss of neuroligin 2, gephyrin, and postsynaptic GABAA receptors, but presynaptic GABA transporters are not involved. Consequently, a loss of GABAergic synapses could contribute to an increase in excitatory synaptic transmission (Shin et al., 2020). Likewise, changes in the expression of NL2 have been shown to result in altered social behavior as well as altered inhibitory synaptic transmission, hence changing the E-I balance. Overexpressing NL2 in the rat hippocampus caused an increase in GAD65, reduced exploration of novel stimuli and resulted in less offensive behavior, suggesting that the disruption of E-I balance in the hippocampus is highly involved in the modulation of social and emotional behavior in those with psychiatric diseases (Kohl et al., 2013).

### Other Microcircuits Associated With Excitatory and Inhibitory Balance

Recent work in schizophrenia patients has identified an altered resting thalamo-cortical connectivity (Klingner et al., 2014). Higher levels of thalamic-sensory-motor connectivity that could predicted symptoms and reduced levels of thalamic-prefrontal-cerebellar connectivity (Anticevic et al., 2014), reduced levels of prefrontal-thalamic connectivity and increased levels of motor/somatosensory-thalamic connectivity (Woodward et al., 2012) and increased levels of somatomotor-thalamic and reduced levels of PFC-thalamic connectivity in both early stage and chronic patients related to controls have been demonstrated (Woodward and Heckers, 2016). Moreover, the increased fronto-parietal control network connectivity in these patients was found to be correlated with the severity of symptoms (Yang et al., 2016), which may relate to an altered E-I balance. Similarly, alterations in cortico-striatal connections as well as functional connectivity between the thalamus and frontal/sensory support the hypothesis of neural disinhibition in schizophrenia (Coyle, 2006). Early stage patients exhibits altered functional connectivity in projections from inhibitory glutamatergic onto dorsal striatum regions (Anticevic et al., 2015). A recent ^1^H-MRS study found an association between higher glutamate levels and greater inferior parietal BOLD signal in schizophrenic patients using an auditory cognitive control task (Falkenberg et al., 2014).

## Consequences of an Excitatory-Inhibitory Imbalance

### Consequences of Abnormal Glutamatergic Neurotransmission

^1^H-MRS studies have found increased concentrations of *in vivo* glutamate in the precommissural dorsal-caudate, the basal ganglia and thalamus in patients with first-episode schizophrenia (De La Fuente-Sandoval et al., 2011), and in the precommissural dorsal-caudate in subjects at ultra-high risk for developing schizophrenia (De La Fuente-Sandoval et al., 2011). Thirteen studies in schizophrenic patients and five studies in high-risk individuals demonstrated increased glutamate and glutamine concentrations in the medial temporal lobe (Merritt et al., 2016). Recently, using a 7-T magnetic field, two *in vivo* glutamate studies on first-episode schizophrenia patients have shown decreased glutamate levels in the ACC (Reid et al., 2019; Wang et al., 2019); however, this finding was only replicated in a study on patients with predominantly negative symptoms (Kumar et al., 2020).

It is noteworthy that altered glutamatergic neurotransmission is related to the brain structure, clinical symptoms, and cognitive impairments of patients with schizophrenia (Tsai et al., 1998a, b). Glutamate concentrations in the brain were found negatively correlated with the inhibitory influence on excitatory neurons in the dorsal ACC of participants with first-episode psychosis (Limongi et al., 2020), and dysfunction in glutamatergic innervation was found in the circuit between the cortex and the medial dorsal thalamus (Sodhi et al., 2011). Similarly, a reduction of plasma GABA/glutamate ratio shows a positive correlation with the improvement of symptoms (Cai et al., 2010). In addition, schizophrenic patients with lifetime auditory verbal hallucinations were found to have exhibited higher levels of glutamate, compared to patients without lifetime auditory verbal hallucinations (Ćurčić-Blake et al., 2017). Bustillo et al. (2011) found that lower glutamate and glutamine levels were positively correlate with overall impaired cognitive function in patients with this illness. Antipsychotic resistance of patients with schizophrenia has been found to be associated with elevated glutamate levels (Demjaha et al., 2014); higher glutamate and glutamine levels in the dorsal ACC was also found in patients with treatment-resistant schizophrenia (Iwata et al., 2019).

### Consequences of Abnormal GABAergic Neurotransmission

Evidence have shown that cortical GABA deficits were resulted from a dysfunction in GABAergic interneuron subpopulations expressing parvalbumin (PV) or somatostatin (SST). Inhibitory interneurons are essential for normal cognitive processing (Tsubomoto et al., 2019), and PV-positive interneurons are implicated in the pathophysiology of schizophrenia (Nguyen et al., 2014; Stansfield et al., 2015; Stedehouder and Kushner, 2017; Ferguson and Gao, 2018a; Perez et al., 2019). It has widely been recognized that working memory is impaired in schizophrenia, with the DLPFC being highly involved. Animal studies have found that heterozygous deficiency of Gad67, a GABA-synthesizing gene primarily in PV-expressing neurons, contributed to the pathophysiology of schizophrenia (Fujihara et al., 2015). Lower PV mRNA expression in the DLPFC (Chung et al., 2018; Tsubomoto et al., 2019), rather than lower density of PV-positive neurons, was found to be involved in schizophrenia (Enwright Iii et al., 2018). However, a selective loss of PV-positive GABAergic interneurons in the mPFC and hippocampus have been found in animal models of schizophrenia treated with *N*-methyl-D-aspartic acid receptor (NMDAR) antagonist (Lewis and Gonzalez-Burgos, 2006; Stansfield et al., 2015). Region-specific knockdown of PV has been shown to result in neuronal and behavioral deficits that is similar to symptoms of schizophrenia (Perez et al., 2019). In addition, a reduction of GABAergic transmission from PV-positive interneurons was found to affect fear and novelty-seeking behaviors, which might be related to the behavioral phenotype of schizophrenia (Brown et al., 2015).

Schizophrenia-associated SST mRNA elevations have been reported in both posterior and anterior regions in schizophrenia subjects (Tsubomoto et al., 2019). Postmortem evidence suggests a region-specific effect of SST neurons in schizophrenia. A study showed that SST knockdown in the ventral hippocampus led to an increase in the regional activity of pyramidal cells and produced downstream effects on dopamine neuron activity in the ventral tegmental area. In contrast, mPFC knockdown of SST did not affect the activity of ventral striatum dopamine neurons; however, it did result in deficits in negative (social interaction) and cognitive (reversal learning) domains (Perez et al., 2019).

Gamma band oscillations are crucial for cognition and have been found to be altered in patients with schizophrenia with the use of electroencephalography (EEG) and magnetoencephalography (MEG) (Gonzalez-Burgos and Lewis, 2012; Jadi et al., 2016). Studies showed that reduced cortical oscillations were associated with reduced inhibition and were considered to mediate important cognitive processes (Lodge et al., 2009; Lewis, 2012). Aberrant spectral power at low and high gamma band frequencies has been found in both first-episode and chronic schizophrenic patients, and reduced gamma band activity showed a correlation with neuropsychological deficits and clinical symptoms in schizophrenic patients (Grent-’T-Jong et al., 2018). Moreover, increased beta and gamma power has been observed in the local circuits of *in vitro* models of schizophrenia; and it has been shown that this hyper-synchronization derived from a loss function of NMDA could be prevented by clozapine (Rebollo et al., 2018). Schizophrenic patients also showed reduced electroencephalography power at 40 Hz, as well as a delayed onset of phase synchronization(Kwon et al., 1999).

The findings discussed above have provided evidence that abnormal functions of the GABAergic and glutamatergic system underlie the excitatory-inhibitory imbalance in schizophrenia (see Table 1).

**TABLE 1:** Summary of evidence for excitatory-inhibitory(E-I) imbalance in schizophrenia.

	Excitatory-inhibitory(E-I) balance	
	Glutamatergic dysfunction	GABAergic dysfunction
**Cerebrospinal fluid (CSF) and Post-mortem studies**	Findings of CSF glutamate levels are inconsistent. Enhanced levels of glutamate receptor antagonist kynurenic acid (KYNA) is consistent.	Lower CSF and plasma GABA levels in schizophrenic patients were first reported. The GABA levels increased with age, duration of illness, and with therapy of long-term neuroleptic.

**Microcircuits**	Increased glutamatergic activity in prefrontal, precommissural dorsal-caudate, basal ganglia, thalamus, and medial temporal lobe, decreased glutamatergic activity in the anterior cingulate cortex were reported.	Increased prefrontal, anterior cingulate cortex, and parieto-occipital cortex GABAergic activity; reduced GABA levels in ACC showed correlation with illness severity were reported.

**Animal models**	Genetic models: DISC1, RELN, CLU3 and ATX, PGC-1α–/–, 22q11.2 1.5 Mb deletion [Df(16)A ± ], NL2 R215H knock-in mouse model of schizophrenia suggested alterations of E-I balance.	

**Pharmacological studies**	Dysfunction of NMDAR could cause schizophrenia-like behaviors in animal models and humans. Antagonists of NMDAR could produce positive, negative and cognitive symptoms in schizophrenia. D-serine and riluzole may be effective in the treatment of negative symptoms.	Dysfunction of ionotropic GABA type A receptors represents a core pathophysiological mechanism underlying cognitive dysfunction in schizophrenia. Lorazepam could exacerbate working memory deficits in schizophrenia. However, flumazenil could ameliorate working memory deficits in schizophrenics.

## Summary and Future Outlook-Where Do We Stand and What Are Future Studies Required for a Better Understanding of This Disease

The relationship of the two opposing actions, excitation and inhibition, could influence difference brain functions, and its aberrance might result in a phenotype deficit. The stability and balance between excitation and inhibition in time and space form the basis of individual phenotypes. Previous studies on E-I balance are mostly limited to the ratio of synapsis, single cells or at the overall level in a certain timescale. However, the E-I balance of the whole brain is a complicated process, which has made this concept multidimensional. The balance status between excitation and inhibition signals is different in various subtypes of excitatory or inhibitory neurons and global circuits on dynamic timescales.

Schizophrenia is a heterogeneous disorder, and biomarkers might help identify its subtypes. The findings of studies related to subtype identification of schizophrenia, such as brain imaging deficit, eye tracking deficit, smell deficit, electrophysiological abnormality, immunity alteration, and genetic and epigenetic abnormalities, are only present in patients with some, rather than all, of the subtypes. These interconnected markers may serve as intermediate phenotypes underlying different subtypes of schizophrenia. It is also speculated that the imbalance between neuronal excitation and inhibition might be the core pathology for schizophrenia. Data from clinical studies in patients with schizophrenia as well as animal models of schizophrenia have provided evidence for the E-I imbalance in schizophrenia. Postmortem studies, CSF assessment and *in vivo* imaging studies also suggested that patients with schizophrenia had altered concentrations of GABA and glutamate, which indicated an alteration in E-I signaling at levels of individual synapses, circuits and networks. The alterations in E-I balance are associated with the severity of clinical symptoms and cognitive dysfunction. Several factors are highly likely to contribute to E-I imbalance, including developmental alterations, as well as alterations at the cell, circuit, network and receptor levels; the variety of factors might be the reason for the heterogeneity in clinical, cognitive, social, and behavioral phenotypes of schizophrenia patients.

Future work is required to further investigate the E-I imbalance in different spaces and dynamic timescales, as well as the consequence of time- and space-specific E-I imbalance in schizophrenia. Although the role of E-I imbalance in schizophrenia has been demonstrated, the role of this imbalance in different subtypes of schizophrenia and the time of onset is still unclear. E-I imbalance happening in various developmental stages (e.g., prenatal, adolescent, and adult stages) may reflect distinct mechanisms (long-term or permanent deficit) and result in different phenotypes. In addition, how could the E-I balance regulation improve clinical symptoms, cognitive deficits, or social-behavioral disturbances in schizophrenia also remains unclear. Future studies may also focus on the identification of biomarkers that might indicate E-I balance disruption in specific circuits and networks, predicts the type of clinical, cognitive, and social-behavioral phenotypes, and indicate which intervention might be effective. These efforts may help reveal new targets for pharmacological approaches and facilitate further development of treatment for the schizophrenia. Moreover, E-I imbalance has been reported in many neuropsychiatric disorders, including autism spectrum disorders, schizophrenia, and depression. However, whether this imbalance is shared between these diseases or a disease-specific alteration still requires further exploration. Therefore, there is still a long way to go with regards to the exploration of etiology, development and selection of treatment, and assessment of prognosis of schizophrenia.

## Author Contributions

YL drafted the manuscript. YL, YN, and WG conceived and designed the review. YL, PO, and LM reviewed the references. YZ, JZ, YN, and WG critically revised the manuscript. All the authors reviewed and approved the final revision.

## Conflict of Interest

The authors declare that the research was conducted in the absence of any commercial or financial relationships that could be construed as a potential conflict of interest.

## Publisher’s Note

All claims expressed in this article are solely those of the authors and do not necessarily represent those of their affiliated organizations, or those of the publisher, the editors and the reviewers. Any product that may be evaluated in this article, or claim that may be made by its manufacturer, is not guaranteed or endorsed by the publisher.
